# Genomic and molecular alterations associated with primary resistance to immune checkpoint inhibitors

**DOI:** 10.1007/s00262-024-03825-z

**Published:** 2024-09-13

**Authors:** Jyoti Malhotra, Subhajyoti De, Kim Nguyen, Percy Lee, Victoria Villaflor

**Affiliations:** 1 City of Hope National Medical Center, 1500 E. Duarte Road, Duarte, CA 91010 USA; 2 Rutgers Cancer Institute of New Jersey, New Brunswick, NJ USA

**Keywords:** Molecular, Genomic, Primary resistance, Immunotherapy

## Abstract

The clinical response to immune checkpoint inhibitors may vary by tumor type and many tumors present with either primary or acquired resistance to immunotherapy. Improved understanding of the molecular and immunologic mechanisms underlying immunotherapy resistance is essential for developing biomarkers and for guiding the optimum approach to selecting treatment regimens and sequencing. This is increasingly important for tumors with primary resistance as effective biomarkers in this setting can guide clinicians about appropriate treatment regimen selection in the first-line setting. Multiple potential biological mechanisms of primary resistance have been proposed but most are yet to be validated in prospective clinical cohorts. Individual biomarkers have poor specificity and sensitivity, and the development of validated and integrated predictive models may guide which patient will benefit from monotherapy versus combination therapy. In this review, we discuss the emerging data identifying the molecular mechanisms of primary resistance to immunotherapy and explore potential therapeutic strategies to target these.

## Introduction

Immunotherapy with immune checkpoint inhibitors (ICIs) targeting the PD-1/PD-L1 and CTLA4 axis has transformed clinical outcomes for patients across several cancer histologies and has become an integral part of standard treatment regimens. Depending on the tumor type, the response to ICIs varies, and many tumors present with either primary or acquired resistance to immunotherapy. There are emerging data on potential mechanisms underlying primary and/or acquired resistance to immunotherapy which is derived mainly from either preclinical studies or secondary correlative analyses from clinical trials. Improved understanding of the molecular and immunologic mechanisms underlying immunotherapy resistance is essential for developing biomarkers and for guiding the optimum approach to selecting treatment regimens and sequencing. This is increasingly important for tumors with primary resistance as effective biomarkers in this setting can guide clinicians about appropriate treatment regimen selection in the first-line setting. Multiple potential biological mechanisms of primary resistance have been proposed: ineffective priming of a T-cell response, lack of tumor recognition due to defective antigen presentation, inability of T cells to penetrate effectively, and the inability of T cells to eliminate tumor cells due to suppression via other checkpoints such as lymphocyte-activation gene 3 (LAG-3) [1]. Tumor-immune evasion and resistance to PD-(L)1 inhibitors can also be mediated by canonical cancer signaling pathways such as Wnt–β-catenin signaling, cell cycle regulatory signaling, and mitogen-activated protein kinase signaling. In this review, we discuss the emerging data identifying the molecular mechanisms of primary resistance to immunotherapy and explore potential therapeutic strategies to target these.

## Defining primary resistance to immunotherapy

The immune evasive measures used by cancer cells are broadly separated into two categories based on the tumor microenvironment: an inflamed T-cell phenotype that suppresses immune activation and a non-inflamed phenotype that passively escapes immune detection [2, 3]. The inflamed phenotype is characterized by tumor infiltration by CD8 + T cells leading to tumor cell cytotoxicity [4]. T-cell activation is a well-regulated process that involves a balance between co-stimulatory and co-inhibitory signals exchanged in the binding of the T-cell receptor (TCR) to the major histocompatibility complex (MHC) peptide complex or the antigen presenting cells (APCs). These antigens, which include tumor-derived neoantigens, are phagocytosed, processed, and presented in the MHC molecules on the cell surface of APCs in recognizable form to train immune cells such as effector T cells, leading to their activation [5]. Two main signals are required for T-cell activation: (I) engagement of MHC-bound antigen on APCs by the TCR, and (II) co-stimulation via CD80/CD86 and CD28 interactions between APCs and T cells. PD-L1/PD-1 interactions counteract this co-stimulation [6, 7]. The PD-1/PD-L1 pathway is a crucial self-tolerance pathway that tumor cells hijack to escape immune elimination and ICIs such as PD-(L)1 inhibitors target this pathway to enable immune mechanisms to target tumor cells [2].

Resistance to ICIs is complex and can present immediately after treatment initiation (primary resistance) or after initial clinical benefit (secondary or acquired resistance) [8]. As per recommendations from the first meeting of the SITC immunotherapy resistance taskforce, a patient who has disease progression after receiving at least 6 weeks (~ two complete cycles of therapy), but no more than 6 months of ICI therapy is considered to have primary resistant disease [8]. Conversely, any patient who experiences progression after demonstrating initial clinical benefit with PD-(L)1 inhibitor is defined as having secondary or acquired resistance. The biological definition of primary resistance to PD-(L)1 inhibitors is defined as the inability of immune cells to mount an antitumor response on initial drug exposure [9]. The rates of primary resistance to immunotherapy vary and range from 35 to 40% for metastatic melanoma [10, 11] and 39% for advanced NSCLC [12]. To date, the clinical factors that have been associated with primary resistance are elevated levels of serum LDH [13], increased tumor burden [14], and lack of PD-L1 expression [15] but it is unclear whether these measures are surrogates for resistance or have a direct mechanistic role [16]. The tumor microenvironment, particularly the presence and activity of T cells, has also been shown to influence resistance [17] with both tumor-extrinsic as well as tumor-intrinsic molecular mechanisms such as lack of T-cell infiltration [18], insufficient neoantigens [19], or absence of an interferon signature [20] identified as being associated with ICI resistance.

## Genomic and molecular mechanisms of primary resistance

Multiple tumor-intrinsic molecular mechanisms have been identified for primary resistance to immunotherapy: lack of T-cell response due to loss of antigen presentation (deletion in beta-2-microglobulin (β2M)), genetic T-cell exclusion (MAPK or PI3K oncogenic signaling, stabilized b-catenin, oncogenic PD-L1 expression), or insensitivity to T cells (mutations in interferon-gamma pathway signaling) [21].

### Deletion, mutation, or loss of heterozygosity in β2M

β2M is associated with the heavy chain of MHC I, and thus, β2M mutations impact MHC I antigen presentation. Without β2M on the tumor cell surface, HLA class I molecules are unstable and unable to present antigen to CD8 + T cells. β2M point mutations, deletions, or loss of heterozygosity (LOH) were detected in 29% of patients (n = 17) with metastatic melanoma progressing on ICI treatment [22]. In two independent cohorts of melanoma patients treated with anti-CTLA4 and anti-PD-1, β2M LOH was enriched threefold in non-responders (~ 30%) compared to responders (~ 10%), and loss of both copies of β2M was found only in non-responders [23]. In β2M gene knockout models in human and murine cell lines, lack of antigen presentation, cancer cell recognition, and cytotoxicity by T cells was observed [23]. Notably, β2M loss does not affect response to ICIs in mismatch repair–deficient colorectal cancer and these tumors have been observed to have increased intratumoral infiltration of CD4 + T cells [24]. This indicates that CD4 + T cells may play a role in ICI resistance associated with β2M loss and CD4 + T-cell-based adoptive therapy approach may be evaluated further in this setting [25].

Several approaches are being explored to target tumors without intact β2M. Bempegaldesleukin (NKTR-214), a prodrug of conjugated IL-2 leads to sustained activation of the IL-2 pathway and leads to a systemic expansion of both CD4 + , CD8 + T, and NK cells [26]. Combination therapy with NKTR-214 and ICI may be synergistic, and the administration of NKTR-214 attenuated anti-PD-1 resistance in β2M knockout tumors and prolonged survival in β2M knockout melanoma mice [27]. However, the clinical program with NKTR-214 was discontinued when the primary endpoint was not reached in the phase III PIVOT-09 and PIVOT-10 trials in RCC and urothelial cancers (NCT03729245, NCT03785925). NK cell-based therapy may also be a promising immunotherapy for ICI-resistant melanoma caused by β2M deficiency [28] as NK cells may be activated to recognize “missing self” [29]. Another potential strategy is to use plasmids or adenoviruses to deliver wild-type human β2M gene into tumor cells to restore tumor cell HLA class I antigen expression [28]. An in vivo study using the Ma-Mel-86b tumor xenograft model in nude mice showed that the intratumoral injection of β2M-carrying adenoviral vectors restored regular HLA class I expression [30]. Based on this design, Allovectin-7 (velimogene aliplasmid) is a bicistronic plasmid DNA encoding two transgene proteins, HLA-B7 and β2M [31]. In a phase I trial, Allovectin-7 enabled the synthesis and expression of intact MHC class I complexes on the tumor cell surface and stimulated T-cell-based immune responses to transfected cells and foreign antigens [32, 33]. In a phase II trial of Allovectin-7 among 133 patients with advanced melanoma [34], the overall response rate (ORR) was 11.8% and the safety profile was acceptable. Subsequently, the phase III Allovectin immunotherapy for metastatic melanoma (AIMM) trial was conducted but reported that responders to Allovectin-7 had significantly shorter overall survival (OS; 18.8 months versus 24.1 months, *P* = 0.491) [35]. This phase III trial included all patients with melanoma and was not limited to tumors with B2M loss or other biomarkers of MHC1 function. The further development of this agent was discontinued. Another approach to target ICI resistance is using radiation therapy (RT). In lung cancer in vivo studies, Wang et al. have demonstrated that localized RT can induce IFN-β production which in turn increases MHC I expression in PD-1 resistant tumor cells and may target PD-1 resistance [36]. Conversely, this radio-sensitization to ICIs is observed only when type I IFN signaling is intact [36].

### Mitogen-activated protein kinase (MAPK) signaling pathway

MAPK directs interactions between tumor cells and the surrounding T-cell infiltrate, downregulates T-cell co-stimulatory molecules, and suppresses the expression of negative immune checkpoints such as PD-L1 and CTLA4 in several cancers [37]. In breast cancer, dysregulation of MAPK pathway has been linked to an immune-silent phenotype associated with poor outcome and treatment resistance [38]. These aberrations include mutations of MAP3K1 and MAP2K4, amplification of KRAS, BRAF, and RAF1, and truncations of NF1 [38]. Activation of the Ras-MAPK pathway correlates with reduced tumor-infiltrating leukocytes (TILs) in a subset of triple negative breast cancer patients who failed to achieve pathologic complete response (CR) after neoadjuvant therapy [39]. The combination of MEK inhibitors and PD-(L)1 inhibitors has been investigated to overcome this resistance. A phase 1 trial (NCT02027961) investigated the combination of durvalumab (anti-PD-L1) with dabrafenib (BRAF inhibitor) and trametinib (MEK inhibitor) in patients with BRAF-mutated melanoma as well as durvalumab and trametinib given concurrently or sequentially in patients with BRAF wild-type melanoma. ORR was 69.2%, 20.0%, and 31.8%, respectively, with improved tumor-immune infiltration in available biopsy samples [40]. Additional trials are currently ongoing. The sequence in which immunotherapy is used with MEK inhibitor may be significant as well. In a phase III trial in metastatic melanoma, immunotherapy with nivolumab and ipilimumab was associated with an ORR of 46%. Conversely, when nivolumab and ipilimumab combination therapy was administered in second line setting in patients who had already received prior therapy with dabrafenib/trametinib, the ORR was lower at 29.6% [41].

### PI3K signaling or loss of PTEN

Signaling through the PI3K/AKT/mTOR pathway contributes to tumorigenesis through several processes such as apoptosis, proliferation, motility, and metabolism. A common way to activate this pathway is through loss of expression of the tumor suppressor PTEN, a lipid phosphatase suppressing the activity of PI3K signaling. Additionally, PTEN represses the expression of immunosuppressive cytokines IL-10, IL-16, and VEGF, by blocking the PI3K pathway [42]. PTEN deletion promotes AKT phosphorylation, thereby promoting PI3K/AKT pathway activation, and ultimately inactivation of T cells.

Peng et al. [43] reported for the first time that loss of PTEN may lead to primary resistance to anti-PD-(L)1 therapy. In preclinical melanoma models, loss of PTEN reduced CD8 + T cells tumor infiltration and impeded T-cell-mediated tumor killing. In an analysis of 135 resected advanced melanoma with regional metastases, tumors with loss of PTEN had lower CD8 + T-cell tumor infiltration as compared to PTEN intact tumors. The loss of PTEN significantly up-regulated the expression of immunosuppressive cytokines VEGF and CCL2, leading to reduced T-cell infiltration in tumors, and suppressed autophagy, thus reducing T-cell-mediated cell death. Loss of PTEN has also been reported to be associated with ICI resistance in uterine leiomyosarcoma [44], as well as in glioblastoma [45].

To target the PI3K-AKT pathway, one study combined PD-1 and PI3K inhibitors in a mouse model of head and neck squamous cell carcinoma [46]. It observed increased survival due to an activated immunostimulatory transcriptional program, enhanced expression of proinflammatory cytokines, and enhanced T-cell cytotoxicity [46]. Another preclinical study with triple CTLA4, PD-1, and PI3K blockade reshaped the tumor microenvironment, enhancing T-cell–mediated tumor regression [47]. Several trials are evaluating the combination of PD-(L)1 inhibitors with PI3K inhibitors (NCT02646748).

### Wnt/β-catenin signaling pathway

Wnt/β-catenin signaling is involved in several cell processes. In melanoma cells in vivo, Wnt/β-catenin signaling prevents the priming of antitumor responses by disrupting the recruitment of dendritic cells expressing basic leucine zipper transcriptional factor ATF-like 3 (BATF3) [48]. In several cancers such as colon cancer, mutations in the destruction complex components (APC, AXIN2, and FAM123B/WTX) or regulators of the receptors/ligand (RNF43/ZNRF3, RSPO2, or RSPO3) components can lead to unchecked Wnt signaling [49]. Furthermore, the conversion of tryptophan into kynurenine is catalyzed by IDO1, which is a transcriptional target downstream of Wnt5A-induced signaling and this metabolic shift promotes the development of regulatory T cells while suppressing effector T-cell activity [50]. Inhibition of this metabolic shift augments the efficacy of anti-PD-1 immunotherapy in a model of BRAF V600E/PTEN − / − mouse melanoma [51].

Spranger et al. [52] used mouse melanoma models to describe how tumor-intrinsic active β-catenin signaling results in T-cell exclusion and ICI resistance. There was also a correlation between activation of the Wnt/β-catenin signaling pathway and absence of a T-cell gene expression signature in tumor samples from patients with metastatic melanoma. Another study analyzing samples from gastric cancer reported that a high β-catenin expression was associated with an absence of CD8 + T-cell infiltration [53]. A large analysis The Cancer Genome Atlas (TCGA) reported Wnt/β-catenin signaling genes were significantly mutated in all colorectal cancer subtypes, and activated Wnt/β-catenin was correlated with the absence of T-cell infiltration [54].

Several drugs inhibiting constitutive Wnt/β-catenin pathway signaling are in clinical development and can be combined with anti–PD-(L)1 therapy to overcome this mode of primary resistance. One study identified serine/threonine-protein kinase PAK4, a Wnt signaling mediator, to be enriched in immunologically cold tumors from patients with melanoma not responsive to anti-PD-1 immune checkpoint blockade [55]. In mouse models, deletion or pharmacological inhibition of PAK4 resulted in reversal of resistance to anti-PD-1 therapy. DKN-01 is a monoclonal antibody neutralizing DKK1, an immune-suppressive protein produced by tumors with Wnt/β catenin activation. A recent phase IIa trial exploring the effect of DKN-01 in combination with chemoimmunotherapy showed encouraging results in patients with advanced gastroesophageal adenocarcinoma [56]. DKB-01 is also under investigation in a phase I/II trial (NCT03645980) as a potential treatment of hepatocellular carcinoma with Wnt/β catenin activation.

### Oncogenic PD-L1 expression

Intrinsic or constitutive PD-L1 overexpression is associated with the presence of multiple molecular mechanisms such as genetic amplification of chromosome 9, which contains the locus of PD-L1 and PD-L2, MYC overexpression, and EGFR or ALK alterations observed in NSCLC [57]. While constitutive PD-L1 signaling exerts a tumor-promoting function through the activation of oncogenic pathways, these tumors are associated with reduced TILs thereby making them “immune-cold” and unlikely to respond to ICIs [57, 58]. In NSCLC, patients with EGFR mutations or ALK rearrangements have very poor response to PD-(L)1 inhibitors. In a retrospective analysis of 58 NSCLC patients treated with PD-(L)1 inhibitors, responses were observed in only 4% of ALK-positive or EGFR-mutant tumors versus 23% of ALK-negative/unknown and EGFR wild-type tumors [59]. PD-L1 expression was observed in 47% and 16% of tumors, respectively, and the PD-L1 expression (≥ 5%) was not concurrently associated with high levels of CD8 + TILs (observed in only 1 pretreatment and 5 resistant EGFR-mutant tumor samples but not observed in any ALK-positive, pre- or post-TKI tissues). This lack of CD8 + TILs and concurrent PD-L1 expression indicates innate PD-L1 expression and underlies the limited effectiveness of PD-(L)1/PD-L1 inhibitors in a majority of EGFR-mutant and ALK-positive NSCLCs [59]. In a study of 336 treatment-naïve EGFR-mutated NSCLC cases, Liu et al. [60] reported low immunogenicity of EGFR-mutated NSCLC by analyzing the TCGA data and an independent validation cohort of patients. In mouse models of EGFRvIII-mutant GBM, CXCL2, and CXCL3 induced local and systemic recruitment of PMN-MDSCs, which correlated with resistance to PD-1 and CTLA4 checkpoint inhibition, CXCR2 antagonism resulted in systemic decrease of PMN-MDSC and enhanced the efficacy of combination immune checkpoint blockade and prolongation of survival [61]. The combination of anti-PD-L1 and EGFR-TKI has been used as a strategy to overcome immunotherapy resistance and yielded a response rate of 43%; however, further development has been halted due to an increased incidence of interstitial lung disease [62]. Amivantamab, an EGFR-MET targeting bispecific in being investigated in combination with anti-PD-1 antibody, cetrelimab in the PolyDamas trial (NCT05908734).

### Interferon-gamma (IFN-γ) signaling pathway

IFN-γ plays an important role in innate and adaptive immunity and demonstrates antiviral, immune-regulatory, and antitumor activity [63, 64]. Upon tumor antigen recognition, T cells produce IFN-γ, which through the IFN-γ receptors (IFNGR1, IFNGR2), the Janus kinases (JAK1 and JAK2) and the signal transducers and activators of transcription (STATs) lead to antitumor effects, such as increased antigen presentation, transporters associated with antigen processing (TAP) and MHC, as well as increased production of chemokines [65]. IFN-γ signaling induces or enhances MHC class I antigen presentation, a process that requires coordinated expression of several genes, including TAP1, TAP2, β2M, and the immunoproteasome genes PSMB8, PSMB9, and PSMB10 [66]. Copy number alterations of the IFN-γ signaling pathway are associated with primary resistance to ICIs with the most coming being genomic loss of key IFN-γ pathway genes such as IFNGR1, IRF-1, JAK2, and IFNGR2, as well as amplification of important IFN-γ pathway inhibitors including SOCS1 and PIAS4 [67]. JAK1/2 loss-of-function mutations are a genetic mechanism of lack of reactive PD-L1 expression and response to IFN-γ, leading to primary resistance to PD-1 blockade therapy. A large analysis using TCGA melanoma dataset reported that 6% and 11% of tumors harbored alterations in JAK1 and JAK2, respectively, and were associated with worse outcomes [68]. The oncogenic activation of MYC in SCLC cells via MYC amplification downregulates JAK2 and impairs IγSGs stimulation by IFNγ [69].

One approach to overcome immunotherapy resistance is to induce a strong IFN response by triggering pattern recognition receptors. Synthetic CpG oligodeoxynucleotide (ODN) agonists of TLR9 are being tested in the clinic in combination with the PD-(L)1 inhibitors. The combination of intratumoral SD101 (a CpG-ODN) with pembrolizumab resulted in antitumor responses in patients with advanced melanoma who were refractory or resistant to prior anti-PD-1 therapy [70]. RNA profiling of tumor biopsies demonstrated increased CD8 + T cells, natural killer cells, cytotoxic cells, dendritic cells, and B cells. Vidutolimod, a TLR9 agonist, was investigated in a phase 1b study (CMP-001-003; NCT03438318) in combination with atezolizumab with and without radiation therapy in patients with advanced NSCLC [71]. The combination was well-tolerated and 15% to 25% of patients had stable disease as the best response. Talimogene laherparepvec (T-VEC) is an oncolytic virus that is FDA approved for the treatment of metastatic melanoma. It preferentially replicates within tumor cells and expresses the cytokine GM-CSF to promote the maturation and activation of APCs in the vicinity and therefore, does not interfere with antigen presentation in infected cells [72]. In combination with anti-PD-1 therapy, T-VEC resulted in ORR of 62% in a phase Ib study [72] in patients with metastatic melanoma. Moreover, 3 of 5 patients with low baseline IFN-γ production had CR, supporting a role for T-VEC in patients without preexisting antitumor-immune responses. SC-43, a SHP-1 agonist that inhibits STAT3, is being investigated in combination with cisplatin in a phase I/II trial for NSCLC (NCT04733521). TTI-101 is a first-in-class, orally bioavailable, selective small molecule that prevents STAT3-mediated transcriptional activity [73] and, a phase I/II study of TTI-101 in monotherapy or in combination with pembrolizumab or atezolizumab–bevacizumab is currently ongoing (NCT05440708).

### Other

In an immunogenomic analysis of TCGA data and clinical trials of anti-PD-(L)1 therapy, 9p21 loss confers “cold” tumor-immune phenotypes [74], characterized by reduced abundance of TILs, particularly, T/B/NK cells, altered spatial TILs patterns, diminished immune cell trafficking/activation, decreased rate of PD-L1 positivity, along with activation of immunosuppressive signaling. Tumors with 9p21 loss exhibited significantly lower response rates to ICIs across eight trials of > 1,000 patients. Among genes mapping to the chromosomal region 9p21.3, CDKN2A was most frequently deleted (13.5%), followed by MTAP (9.3%). In addition to homozygous deletion (HD), 9p21 LOH due to hemizygous deletion of CDKN2A and MTAP was observed in 24.6% and 27.8% of cancers, respectively. In addition, although LOH of 9p21 did not lead to massive changes in CDKN2A/MTAP expression, it conferred significantly shorter OS in comparison with tumors with diploid/wild-type 9p21. The association between CDK4 gain (on 12q14.1 loci) and primary resistance to anti-PD-(L)1 therapy was validated in 85 patients with advanced melanoma (*P* < 0.05) [75]. RNA-Seq analysis of CDK4-normal cell lines and CDK4-normal tumors showed altered transcriptional output in TNFα signaling via NF-κB, inflammatory response, and IFNγ response gene set. In addition, CDK4/6 inhibitor (palbociclib) treatment increased PD-L1 protein levels and enhanced efficacy (*P* < 0.05). The phase II NEWFLAME trial investigated the combination of nivolumab, abemaciclib, and endocrine therapy (fulvestrant or letrozole) in patients with hormone receptor positive metastatic breast cancer [76]. While the ORR was 4% to 54%, there was a high incidence of severe immune-related adverse events, such as hepatotoxicity thus limiting further investigations.

The mechanism of resistance for several genomic predictors of ICI resistance remains unclear. STK11/LKB1 and KRAS co-mutated NSCLC is a distinct subgroup with primary resistance to ICIs. In this cohort of patients, the response rates to PD-(L)1 inhibitors were only 7.4% in the Stand Up To Cancer (SU2C) cohort (174 patients) with KRAS-mutant NSCLC and 0% in patients treated with nivolumab in the CheckMate-057 phase III trial [77]. In KRAS-mutant murine LUAC models, STK11/LKB1 loss promoted PD-(L)1/inhibitor resistance [77], possibly due to altered cytokine/chemokine milieu, metabolic restriction of effector T cells, or impaired antigenicity. KEAP1 loss has been identified to lead to innate ICI resistance in NSCLC [78] likely by suppressing CD103 DC-mediated CD8 T-cell immunity [78]. CB839, a glutaminase inhibitor, may be a strategy [79]. In an analysis of 155 patients with stage IV solid tumors, all six tumors with MDM2/MDM4 amplification were noted to have primary resistance as well as hyper-progression on ICIs. Two of 10 patients with EGFR alterations were also hyper-progressors [80].

One genomic biomarker predictive of response to immunotherapy is tumor mutational burden (TMB), which is a measure of the number of mutations in a cancer [81]. Several studies have reported a relationship between TMB and immunotherapy efficacy with a higher TMB being associated with increased responsiveness to ICIs [82–84]. Since neoantigens are related to the number of mutations, the higher the TMB, greater is the chance that some of the neoantigens may be immunogenic [83, 85]. However, there is variability in how well TMB can predict response to ICIs for a patient due to other factors that may influence presence or absence of immunogenic neoantigens as well as the impact of histology on TMB [83, 85, 86]. There is also currently no consensus in the TMB cutoff to be used for patient stratification [81]. Another factor that influences ICI resistance is the clonality of neoantigens [87]. Genomic intratumor heterogeneity, which results in subclonal mutations and neoantigens, and high subclonal TMB is associated with an ineffective antitumor response [87, 88]. Conversely, clonal TMB is a stronger predictor of ICI responsiveness than total TMB [88, 89].

Cancer cell-intrinsic mechanism of primary resistance to immunotherapy has been outlined by the expression of a certain set of genes that are enriched in tumors from patients who did not respond to anti-PD-1 therapy [90]. This set of genes was termed innate anti-PD-1 resistance signature (IPRES) and includes regulatory genes such as epithelial–mesenchymal transition-related genes (AXL, WNT5A, ROR2, TWIST2, FAP, and TAGLN), VEGF pathway genes (IL-10, VEGFA, and VEGFC), and macrophage chemotaxis genes (CCL2, CCL7, CCL8, and CCL13) [90]. Spatial interplay between tumor and immune microenvironment influences intercellular signaling, immune recognition, and resistance to immunotherapy [91]. Spatial transcriptomics and multiplexed quantitative immunofluorescence have made it possible to annotate localization of tumor and immune cells with precision [92], which is an emerging tool to understand molecular mechanisms of primary resistance in the spatial context of tumor microenvironment. In a cohort of NSCLC patients, multiplexed tissue imaging identified enrichment of Tregs in non-responders, and these were localized to stromal and peripheral tumor margins. Spatial phenotyping of cytokine signatures in head and neck cancer identified areas of abnormal CXCL9 and CXCL10 expression associated with resistance to immunotherapy [93]. A multi-institutional study reported that spatial distribution of T cells and T-cell exhaustion marker expression predict outcomes with ICIs in NSCLC [94].

### Future and conclusions

**Fig. 1:**
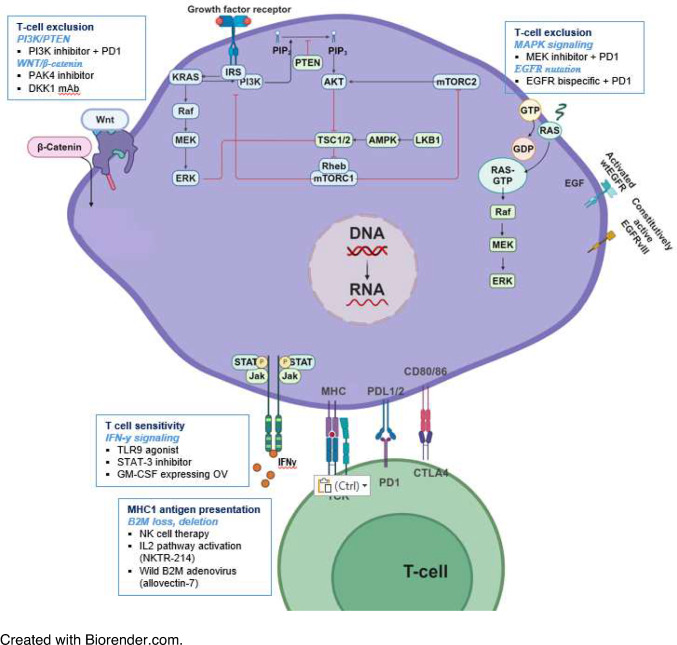
Genomics and molecular mechanisms of primary immunotherapy resistance and current strategies under investigation to overcome resistance. Created with Biorender.com

Several genomic predictors of primary response to ICIs have been identified but most are yet to be validated in prospective clinical cohorts. Further elucidation of these molecular mechanisms is key to developing risk scores as well as treatment strategies. Individual biomarkers have poor specificity and sensitivity, and the development of a validated and integrated predictive model will guide which patient will benefit from monotherapy versus combination therapy. This approach can also be applied to tumors with acquired resistance as some of the molecular alterations driving primary ICI resistance have also been noted for acquired resistance such as JAK2 or β2M mutations [95]. There are several potential approaches to target these mechanisms of primary resistance that have been tested but clinically significant benefit is yet to be seen as these trials have not been in biomarker-selected patient populations. Integration of genomic and other predictors of primary resistance in selecting patients for future trials aimed at overcoming ICI resistance is essential (Fig. 1 and Table 1).

**Table 1:** Completed and ongoing trials targeting genomic predictors of primary immunotherapy resistance

Molecular mechanism	Genomic alterations	Agent	Drug class/mechanism	Completed trials	Ongoing trials
Antigen presentation					
Beta-2-microglobulin related MHC1 antigen presentation	Deletion, point mutation, LOH	Bempegaldesleukin (NKTR-214)	Sustained activation of the IL-2 pathway	NCT03785925 NCT04969861 NCT03435640 NCT03635983	Clinical program discontinued
		NK cell therapy	Recognize “missing self”		NCT05334329
		Allovectin-7 (velimogene aliplasmid)	Intratumoral injection of wild-type B2M-carrying adenoviral vectors	NCT00395070 NCT00050388 NCT00044356	Clinical program discontinued
T-cell exclusion					
MAPK oncogenic signaling	Mutations in MAP3K1, MAP2K4, NF1 truncation	Trametinib or binimetinib	MEK inhibitor (+ PD(L)1 inhibitor)	NCT02027961 NCT02902042 NCT03374254	NCT05440942 NCT03991819
PI3K oncogenic signaling	PTEN loss	Inavolisib or TOS-358	PI3K inhibitor (± PD(L)1 inhibitor)	NCT02646748 NCT04193293	NCT05683418 NCT04551521
Wnt/β-catenin signaling	Mutations in APC, AXIN2, WTX, ZNRF3	PF-03758309 or ATG-019	PAK4 inhibitor	NCT00932126	NCT04281420
		DKN-01	monoclonal antibody neutralizing DKK1	NCT01457417 NCT02013154	NCT04166721 NCT05761951
Oncogenic PD-L1 expression	EGFR mutation, ALK rearrangement (NSCLC)	Osimertinib + durvalumab	TKI + PD(L)1 inhibitor	NCT02143466	Not explored further due to side effects
		Amivantamab	EGFR-MET bispecific + PD(L)1 inhibitor		NCT05908734
T-cell sensitivity					
IFN-y pathway signaling	Loss of IFNGR1, IRF-1, JAK2, IFNGR2 SOCS1 and PIAS4 amplification JAK1/2 loss MYC amplification	SD101 or CMP-001 (Vidutolimod)	TLR9 agonist	NCT02521870 NCT03438318	NCT01042379 NCT05445609
		T-VEC	Oncolytic virus expressing GM-CSF	NCT04068181 NCT03256344	NCT02965716
		SC-43 or TTI-101	STAT3 inhibitor		NCT03443622 NCT05440708
Other					
	CDKN2A loss	Abemaciclib + nivolumab	CDK4/6 inhibitor + PD(L)1 inhibitor	JapicCTI-194782	
	STK11 or KEAP1 mutation	CB839	Glutaminase inhibitor		NCT04250545
	MDM2/MDM4	Brigimadlin (BI 907828)	MDM2-p53 antagonist (± PD(L)1 inhibitor)		NCT03964233 NCT05512377

## Data Availability

No datasets were generated or analyzed during the current study.
